# [Retracted] miR-215 promotes epithelial to mesenchymal transition and proliferation by regulating LEFTY2 in endometrial cancert

**DOI:** 10.3892/ijmm.2026.5877

**Published:** 2026-06-02

**Authors:** Xiaoxu Gao, Yan Cai, Ruifang An

Int J Mol Med 42: 1229-1236, 2018; DOI: 10.3892/ijmm.2018.3703

Following the publication of this paper, it was drawn to the Editor's attention by a concerned reader that western blot data shown in Fig. 1C on p. 1232 were strikingly similar to data in a paper that had previously been published in the journal *Nature Cell Biology* that was written by different authors at different research institutes. Upon performing an independent analysis of the data in this paper in the Editorial Office, it also came to light that the cellular images featured in Fig. 6B on p. 1233 had also previously appeared in another article published in the journal *Science* that was similarly written by different authors at different research institutes.

Given that the abovementioned data had already been published before the receipt of this paper at *International Journal of Molecular Medicine*, the Editor has decided that this paper should be retracted from the Journal. The authors were asked for an explanation to account for these concerns, but the Editorial Office did not receive a reply. The Editor apologizes to the readership for any inconvenience caused.

